# Stress exposure, stress responses, and short-term outcomes in very preterm neonates: a national cohort study

**DOI:** 10.1007/s00431-026-06765-1

**Published:** 2026-02-11

**Authors:** Judith A. ten Barge, Naomi J. Meesters, Manon Benders, Anton H. van Kaam, Bertrand D. van Zelst, Henriette van Zanten, Christ-jan van Ganzewinkel, Maria Luisa Tataranno, Frank A. B. A. Schuerman, Chris H. P. van den Akker, Marlou M. A. Raets, Willem P. de Boode, Peter H. Dijk, Kirsten S. Muller, Irwin K. M. Reiss, Sjoerd A. A. van den Berg, Sinno H. P. Simons, Gerbrich E. van den Bosch

**Affiliations:** 1 Department of Neonatal and Pediatric Intensive Care, Division of Neonatology, Erasmus MC - Sophia Children’s Hospital, Rotterdam, The Netherlands; 2 Department of Neonatology, University Medical Center Utrecht, Wilhelmina Children’s Hospital, Utrecht, The Netherlands; 3 Department of Neonatology, Emma Children’s Hospital, Amsterdam UMC, Amsterdam Reproduction and Development Research Institute, University of Amsterdam, Amsterdam, The Netherlands; 4 Department of Internal Medicine, Erasmus MC, Rotterdam, The Netherlands; 5 Department of Neonatology, Leiden University Medical Center, Leiden, The Netherlands; 6 Department of Neonatology, Máxima Medical Centre, Veldhoven, The Netherlands; 7 Department of Neonatology, Isala Clinics, Zwolle, The Netherlands; 8 Department of Neonatology, Maastricht University Medical Center, MosaKids Children’s Hospital, Maastricht, The Netherlands; 9 Department of Neonatology, RadboudUMC Amalia Children’s Hospital, Nijmegen, The Netherlands; 10 Department of Neonatology, Beatrix Children’s Hospital, University Medical Center Groningen, Groningen, The Netherlands; 11 Department of Child and Adolescent Psychiatry & Psychosocial Care, Emma Children’s Hospital, Amsterdam UMC, University of Amsterdam, Amsterdam Reproduction and Development, Amsterdam Public Health, Amsterdam, The Netherlands; 12 Department of Clinical Chemistry, Erasmus MC, Rotterdam, The Netherlands

**Keywords:** Premature infant, Neonatal Intensive Care, Stress, Cortisol

## Abstract

Very preterm neonates admitted to the neonatal intensive care unit (NICU) face daily stressors that activate their hypothalamic–pituitary–adrenal (HPA) axis, triggering cortisol release and potentially affecting their outcomes. We examined the relationship between NICU stressors, urinary cortisol levels, and short-term outcomes in very preterm neonates. This study was part of a multicenter cohort study (HIPPO) including 446 neonates (gestational age < 29 weeks) across all Dutch NICUs. Data on daily stress exposure for the first 28 days and outcomes were prospectively collected, along with urine samples on postnatal days 8 (T1) and 28 (T2) to determine cortisol levels as markers of the stress response. Urinary samples were available for 391 (88%) neonates. In general, the most preterm born neonates had the highest cortisol levels, though highly variable with similar stress exposure. At T1, cortisol levels rose with increasing stress exposure in neonates born after gestational ages ≥ 27 weeks but decreased in the most preterm. At T2, cortisol levels tended to rise with stress exposure across all gestational ages. Though cortisol levels were higher in neonates that died or developed intraventricular hemorrhage, bronchopulmonary dysplasia or retinopathy of prematurity, cortisol was not independently associated with any of these outcomes.

*Conclusion*: High cortisol levels are linked to neonatal morbidity and mortality, but not independently, since urinary cortisol levels are moderated by gestational age. To protect preterm infants’ future, it is key to understand their responses to stress in order to develop and evaluate stress protection strategies.

**Table:** 

**What is known:** • *Very preterm neonates in the neonatal intensive care unit (NICU) are exposed to high levels of stress on a daily basis*. • *Stress exposure and altered cortisol responses in very preterm neonates have both been linked to adverse outcomes*.	
**What is new:** • *This study reveals gestational age-dependent effects of stress exposure on very preterm neonates’ cortisol levels*. • *In general, high cortisol was associated with morbidity and mortality*. • *This study provides insight in hypothalamic–pituitary–adrenal axis development and highlights the importance of reducing stress*.

**Supplementary Information:**

The online version contains supplementary material available at 10.1007/s00431-026-06765-1.

## Introduction

Preterm neonates admitted to a neonatal intensive care unit (NICU) are exposed to various sources of stress. First, they undergo stressful interventions such as mechanical ventilation, nursing care, and daily diagnostic procedures (e.g., heel lances), experiencing an average of over 10 painful procedures per day [1]. Second, various conditions, including sepsis, necrotizing enterocolitis, and postoperative pain, can cause prolonged stress. Third, the NICU environment, with its lights, noises, and separation from parents, forms an additional source of stress [2].

These stressors evoke a response to restore homeostasis, involving activation of the autonomic nervous system and the hypothalamic–pituitary–adrenal (HPA) axis [3]. The HPA axis releases the stress hormone cortisol, mobilizing energy reserves required during the ‘fight or flight’ response. HPA axis functioning develops early in the second trimester of pregnancy, with basal cortisol levels increasing with gestational age and circadian rhythm appearing approximately one month after birth or term equivalent age [4, 5]. In the face of stressors, it is vital to mount an appropriate cortisol response, and an inability to do so has been associated with increased morbidity and mortality in preterm neonates [6–11].

However, stress exposure itself has also been linked to adverse outcomes, both in the short term (e.g., increased risk of cerebellar hemorrhage) and in the long-term (e.g., impaired cognitive and behavioral development) [12]. High cortisol levels may predict or even contribute to these adverse effects. Repeated stress exposure – also known as allostatic load – early in life may lastingly alter HPA axis function, potentially mediating neurodevelopmental consequences of neonatal stress [13].

Previous studies assessing neonatal stress and cortisol levels have found contradictory results. Measures of disease severity and stress exposure have been related to increases, decreases or no change in cortisol levels [14–24]. This discrepancy may reflect maturational differences between study populations, as (very) preterm neonates’ ability to produce cortisol is limited [25].

The effect of neonatal stress on cortisol levels and its impact on neonatal morbidity thus remains unclear. This study aimed to (1) elucidate the impact of neonatal stress on urinary cortisol levels in very preterm neonates and (2) determine the association between urinary cortisol levels and short-term outcomes.

## Methods

### Study design and sample

This study was part of the Happiness for Improvement of Premature and Parental Outcomes (HIPPO) study, a prospective multicenter cohort study including neonates born before 29 weeks in all level III/IV NICUs in the Netherlands (July 1, 2020 – March 1, 2022). A total of 446 neonates were included and the primary results on neonatal stress exposure have been published previously [26]. In this secondary analysis of HPA axis function, neonates with at least one urinary sample were included. In accordance with the Dutch law on human research, the Erasmus MC medical ethics committee waived the need for formal approval of this study due to its non-interventional nature (MEC-2019–0574). Parents provided written informed consent.

### Data collection

Neonates and parents were prospectively followed from birth until the 28th day of life, or earlier in case of death or NICU discharge. Neonatal stress exposure was assessed daily using the NeO-stress score, a validated measure developed by our research group that quantifies cumulative stress exposure by incorporating the number and intensity of stressors [27]. The NeO-stress instrument was developed by means of two consecutive questionnaires completed by NICU professionals across the Netherlands. First, relevance of potentially stressful procedures was rated and content validity per item calculated. Second, the stressfulness of the most relevant items was scored in order to obtain a severity index. The NeO-stress instrument consists of 38 stressful items and the daily NeO-stress score score is calculated by multiplying the frequency of each stressor by its severity index.

Urine was collected from cotton gauze pads in diapers around day 8 (T1, day 8–11) and day 28 (T2, day 28–31) of life to enable noninvasive assessment of corticosteroid profiles. Urine samples were stored at −20 °C until analysis. For participants with multiple samples at one time point, only the first within the designated period was used.

Short-term outcomes collected prospectively included the core neonatal outcomes mortality and incidence of intraventricular hemorrhage (IVH; stage ≥ I), bronchopulmonary dysplasia (BPD; ≥ 28 cumulative days of supplemental oxygen at 36 weeks postmenstrual age) necrotizing enterocolitis (NEC; Bell’s stage ≥ II), and retinopathy of prematurity (ROP; stage ≥ II) at term-equivalent age [28]. Data on antenatal and postnatal corticosteroid treatment were retrospectively extracted from medical records.

### Corticosteroid analyses

Urinary steroids were measured in an ISO15189 accredited clinical laboratory. An 8-point calibration curve was prepared in phosphate-buffered saline with 2.5% methanol for cortisol, cortisone, cortisol-sulfate and cortisol-glucuronide. Frozen calibrators, controls and patient urine samples were thawed, mixed, and centrifuged. Twenty-five µL of each sample was transferred to 96-well plates and diluted 20-fold with 45% methanol containing labeled isotopes as internal standard for all steroids. Samples were analyzed via liquid chromatography-tandem mass spectrometry (LC–MS/MS) using a Xevo TQ-S mass spectrometer (Waters, The Netherlands) with an Acquity UPLC and HSS T3 1.8 µm column. An 8-min linear gradient of MilliQ water and methanol (55:45 to 46:54) with 2 mmol/L ammonium acetate and 0.1% formic acid was run at 0.30 mL/min at 50 °C. Data were analyzed using MassLynx 4.1, identifying cortisol, cortisone, cortisol-glucuronide, and cortisol-sulphate by relative retention times and ion ratios. Steroids were quantified using linear calibration series corrected by the internal standard, with a detection limit of 5 nmol/L for all steroids.

### Statistical analyses

Descriptive statistics were presented as mean (standard deviation), median (interquartile range), or number (%), as appropriate. Shapiro–Wilk assessed normality. Background characteristics of the total HIPPO cohort and those included in this analysis were compared with unpaired t-tests, Mann–Whitney U tests, and Chi-square tests, as appropriate. A *p*-value below 0.05 was considered statistically significant. Analyses were conducted in RStudio (v2023.12.0) (R Core Team, Vienna, Austria).

#### Description of urinary glucocorticoid profiles

Glucocorticoid concentrations at T1 and T2 were compared with Mann–Whitney U tests, and Spearman correlations between glucocorticoids were calculated. Cortisol-to-cortisone ratios were calculated to assess the balance between 11β-hydroxysteroid dehydrogenase type 1 (11β-HSD1, converting cortisone to cortisol) and 11β-hydroxysteroid dehydrogenase type 2 activity (11β-HSD2, converting cortisol to cortisone). To allow analyses, cortisol values below the limit of detection (5 nmol/L) were replaced with half the detection limit. Primary analyses focused on cortisol as the biologically active metabolite, with data on cortisone presented to reflect 11β-HSD2 activity. Cortisol metabolites cortisol-sulphate and cortisol-glucuronide (formed through hepatic conjugation) were reported for completeness but excluded from further analyses because these were considered less relevant to our research questions, and cortisol-glucuronide measurement often failed.

#### Factors associated with urinary cortisol levels

Linear regression analyses evaluated the association between cumulative NeO-stress score (summed scores on days preceding urine sampling) and cortisol levels, both at T1 and T2. Since cortisol levels were not normally distributed, transformations (logarithmic, square root, cubic, inverse, Box-Cox) were applied and the transformation that produced the most normal distribution was selected. Extreme cortisol outliers > 1000 nmol/L were excluded.

Interactions between cumulative NeO-stress score and both gestational age at birth and sex were added to examine effect modification, and quadratic terms to evaluate nonlinear effects. Likelihood ratio tests assessed model improvement from interaction and nonlinear terms, with significant terms retained based on Wald tests. Models were adjusted for potential confounders [21, 29], including sex, gestational age, birth weight Z-score (Fenton 2013), maternal chorioamnionitis, Caesarean section, type of NICU (single room/open bay), mechanical ventilation, inotropic support, and antenatal and postnatal steroid exposure.

#### Associations between cortisol levels and neonatal outcomes

For each of the outcomes (mortality, IVH, BPD, NEC, and ROP) assessed at term-equivalent age, urinary cortisol levels were compared between neonates with and without the outcome using Mann–Whitney U tests. Moreover, logistic regression analyses evaluated associations between cortisol levels and these outcomes, adjusting for clinical confounders (including sex, gestational age at birth, type of NICU, birthweight Z-score, NEC, focal intestinal perforation, inotropic support, mechanical ventilation, and antenatal corticosteroid treatment) and considering interactions between cortisol level and gestational age. For BPD, stratified regression analyses were also conducted in neonates with and without postnatal corticosteroid treatment.

## Results

Of the 446 HIPPO participants, at least one urine sample was available for 391 (88%) participants (Supplementary Fig. 1). Samples were available at both T1 and T2 for 191 (49%) neonates, only at T1 for 169 (43%), and only at T2 for 31 (8%), resulting in 582 samples (360 (62%) at T1, 222 (38%) at T2). Background characteristics did not differ significantly from the full HIPPO cohort (Table 1).

**Table 1:** Background characteristics of the entire HIPPO cohort and the subset included in the present analysis (*N* = 391 participants)

	HIPPO cohort (*N* = 446)	This study (*N* = 391)	*P*-value*
Gestational age (weeks)	27.3 (26.3–28.3)	27.3 (26.3–28.3)	0.91
Birth weight (grams)	950 (780–1130)	950 (780–1123)	0.87
Birth weight Z-score	3.0 (−3.0; 8.0)	3.5 (−3.0; 8.0)	0.96
Male sex	261 (59%)	229 (59%)	0.99
Twin/triplet	125 (28%)	109 (28%)	1.00
Cesarean delivery	238 (53%)	217 (55%)	0.58
Days in study	28 (18–28)	28 (20–28)	0.14
Apgar score at 5 min	8 (7–9)	8 (7–9)	0.87
Intubated	51 (11%)	48 (12%)	0.78
Inotropic support	81 (18%)	69 (18%)	0.92
Antenatal corticosteroids			
Complete Incomplete None Unknown	267 (60%) 143 (32%) 30 (7%) 6 (1%)	231 (59%) 126 (32%) 28 (7%) 6 (2%)	0.96
Postnatal corticosteroids			
Any	101 (23%)	86 (22%)	0.89
Hydrocortison	77 (17%)	63 (16%)	0.72
Dexamethason	36 (8%)	32 (8%)	0.94
Focal intestinal perforation Unknown	19 (4%) 4 (0.9%)	15 (4%) 3 (0.8%)	1.00
Necrotizing enterocolitis Stage II Stage III Unknown	43 (10%) 22 (51%) 19 (44%) 2 (5%)	37 (9%) 18 (49%) 17 (46%) 2 (5%)	1.00
Bronchopulmonary dysplasia Grade I (mild) Grade II (moderate) Grade III (severe) Unknown	149 (33%) 62 (42%) 18 (12%) 58 (39%) 11 (7%)	141 (36%) 56 (40%) 17 (12%) 58 (41%) 10 (7%)	0.59
Retinopathy of prematurity Stage II Stage ≥ III Unknown	81 (18%) 47 (58%) 32 (40%) 2 (2%)	78 (20%) 46 (59%) 31 (40%) 1 (1%)	0.78
Intraventricular hemorrhage Grade I Grade II Grade III IVH with venous infarction	132 (30%) 42 (32%) 58 (44%) 21 (16%) 11 (8%)	120 (31%) 39 (33%) 54 (45%) 17 (14%) 10 (8%)	0.85
Died	49 (11%)	33 (8%)	0.25

Values are presented as median (IQR) or number (%)

### Description of urinary glucocorticoid profiles

Urine samples were collected at a median (IQR) postnatal age of 8 (7–8) days at T1 and 27 (27–28) days at T2, with median cortisol values of 21.5 nmol/L (IQR 9.0–51.0, range 2.5–13496) at T1 and 6.0 nmol/L (IQR 2.5–13.0, range 2.5–2844) at T2. Cortisol and cortisone levels were significantly higher at T1 compared with T2 (*p* < 0.001), while cortisol-glucuronide and cortisol-sulphate levels did not differ significantly (Supplementary Fig. 2). Paired comparisons in neonates with urine samples at both T1 and T2 yielded similar results. Spearman’s correlations between the glucocorticoids were 0.64 for cortisol and cortisone, 0.64 for cortisol and cortisol-sulphate, and 0.59 for cortisone and cortisol-sulphate.

**Fig. 1:**
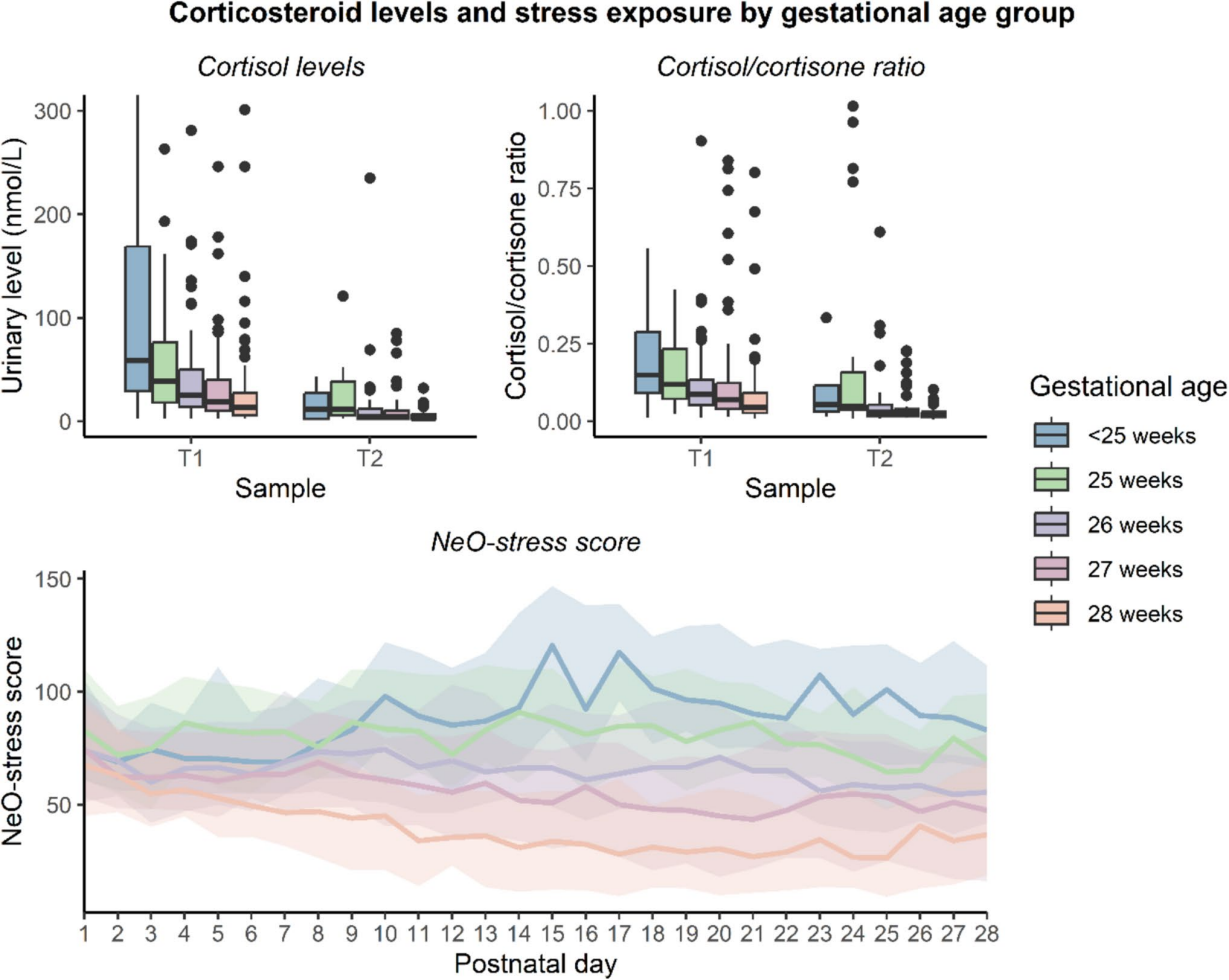
Cortisol levels, cortisol/cortisone ratios and NeO-stress scores by gestational age. To improve clarity, cortisol measurements above 300 nmol/L (*n* = 37) and cortisol/cortisone ratios above 1 (*n* = 24) were omitted from the figure

Figure 1 shows neonates’ cortisol levels and cortisol/cortisone ratios at T1 and T2, along with longitudinal NeO-stress scores (and interquartile range), by gestational age. Cortisone levels generally exceeded cortisol levels in all gestational age groups, even in those with undetectably low cortisol levels (Supplementary Fig. 3). Supplementary Fig. 4 shows the correlation between average NeO-stress scores and cortisol levels at both time points. Neonates born after a gestational age of ≥ 26 weeks exhibited weak positive correlations between average NeO-stress score and urinary cortisol level (correlation coefficients ranging from 0.08 to 0.20). In neonates born before a gestational age of 26 weeks, cortisol levels varied widely despite similar NeO-stress scores.

### Factors associated with urinary cortisol levels

#### Neonatal and maternal characteristics

A higher gestational age was associated with lower cortisol levels at both time points (Table 2). Postnatally administered hydrocortisone was associated with increased cortisol levels at T2, while dexamethasone treatment was linked to decreased cortisol levels at both time points. Single room NICU and inotropic support were associated with higher cortisol levels at T2.

**Table 2:** Linear regression analyses: association between neonatal characteristics and urinary cortisol levels at T1 and T2

	Cortisol levels* at T1			Cortisol levels* at T2		
	*β*	*95% CI*	*P-value*	*β*	*95% CI*	*P-value*
Intercept	12			2.5		
Female sex	0.0065	−0.19; 0.20	0.95	−0.065	−0.15; 0.024	0.15
Gestational age at birth	−0.36	−0.54; −0.18	** < 0.001**	−0.061	−0.10; −0.021	**0.003**
Maternal chorioamnionitis	0.11	−0.24; 0.46	0.54	−0.033	−0.19; 0.12	0.68
Cesarean delivery	−0.049	−0.25; 0.15	0.64	0.066	−0.023; 0.16	0.15
Single room NICU	−0.040	−0.27; 0.19	0.73	0.11	0.0044; 0.21	**0.04**
Antenatal steroid course: complete	0.086	−0.32; 0.49	0.67	−0.046	−0.21; 0.11	0.57
Antenatal steroid course: incomplete	−0.089	−0.50; 0.32	0.67	0.032	−0.13; 0.20	0.70
Birth weight Z-score	0.0081	−0.0028; 0.019	0.15	−0.00061	−0.0053; 0.0041	0.80
Intubated	0.15	−0.16; 0.46	0.33	−0.061	−0.19; 0.064	0.34
Inotropic support	0.18	−0.12; 0.48	0.24	0.12	0.0081; 0.23	**0.04**
Cumulative NeO-stress score	−0.0028	−0.0051; −0.00036	**0.02**	0.000062	−0.0000055; 0.00013	0.07
Cumulative NeO-stress score: gestational age at birth	0.00010	0.000016; 0.00019	**0.02**			
Cumulative hydrocortisone dose	0.0079	−0.011; 0.027	0.41	0.015	0.0085; 0.022	** < 0.001**
Cumulative dexamethasone dose	−0.20	−0.40; −0.0069	**0.04**	−0.14	−0.22; −0.065	** < 0.001**

*Cortisol levels were transformed with a Box-Cox transformation

At T1, 11 cortisol values > 1000 nmol/L and at T2, 9 values > 1000 nmol/L were excluded

#### Stress exposure

**Fig. 2:**
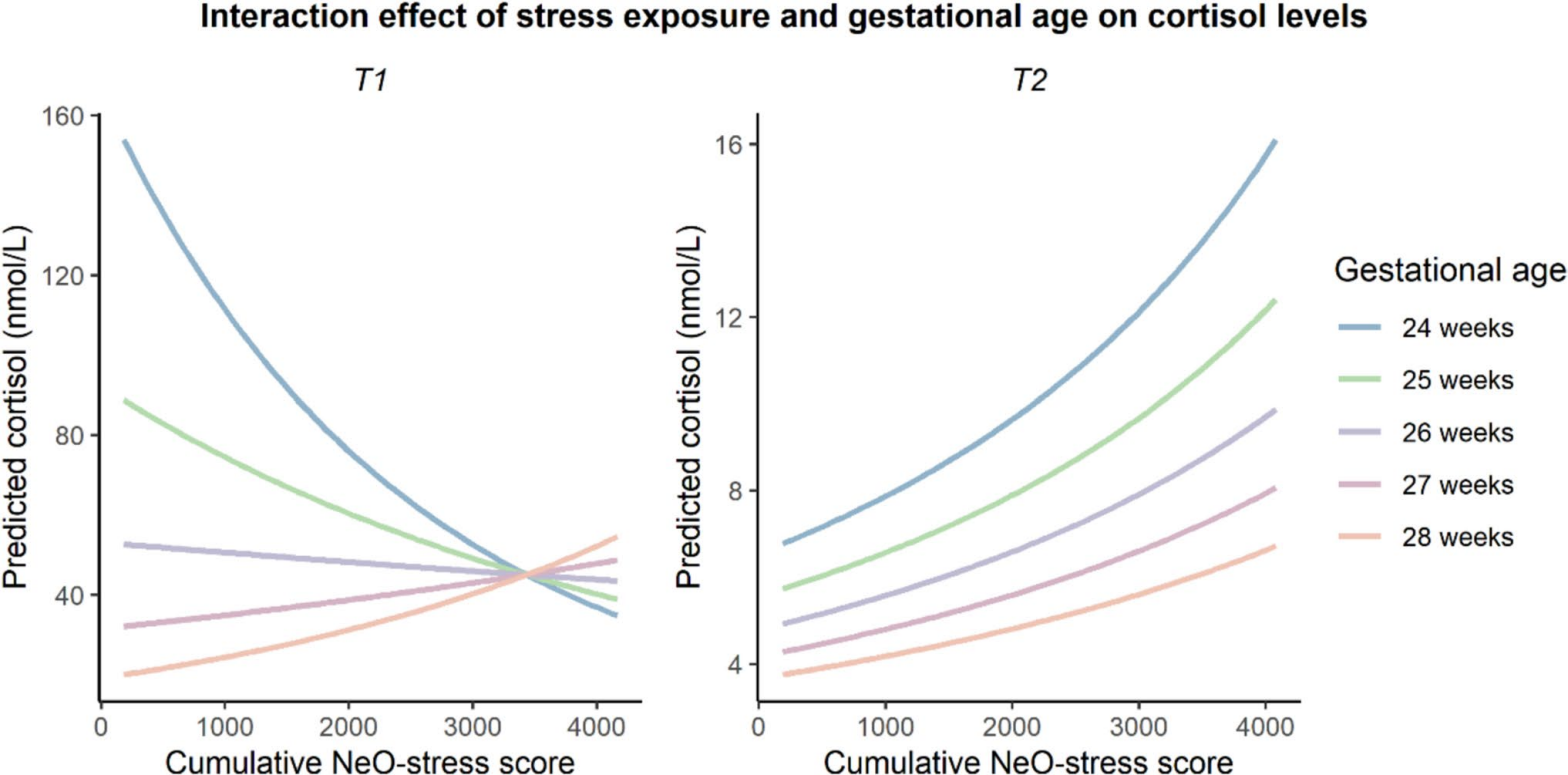
Effect plot illustrating the interaction between cumulative stress exposure and gestational age on predicted cortisol levels. Cortisol values were modelled after Box-Cox transformation but are shown on the original scale for interpretability. The curved lines result from back-transformation rather than significant nonlinear effects. Birth weight Z-score, cumulative hydrocortisone dose, and cumulative dexamethasone dose were set to their population mean. Sex was set to male, antenatal corticosteroid course was set to complete, inotropic support and intubation to 'yes', maternal chorioamnionitis to 'no', birth mode to Caesarean section, and NICU type to open bay

At T1, the association between cumulative stress exposure and cortisol levels was significantly moderated by gestational age (Table 2). In the most preterm born neonates (gestational age < 27 weeks), cumulative NeO-stress score was negatively associated with cortisol levels at T1, while more mature neonates displayed a positive association (Fig. 2). At T2, this interaction between stress exposure and gestational age was not significant (*p* = 0.74) and therefore not included in the model, and cortisol levels tended to increase with stress exposure regardless of gestational age (Table 2). Interactions between stress exposure and sex were not significant, nor were nonlinear terms.

### Associations between cortisol levels and neonatal outcomes

#### Comparisons of cortisol levels

**Fig. 3:**
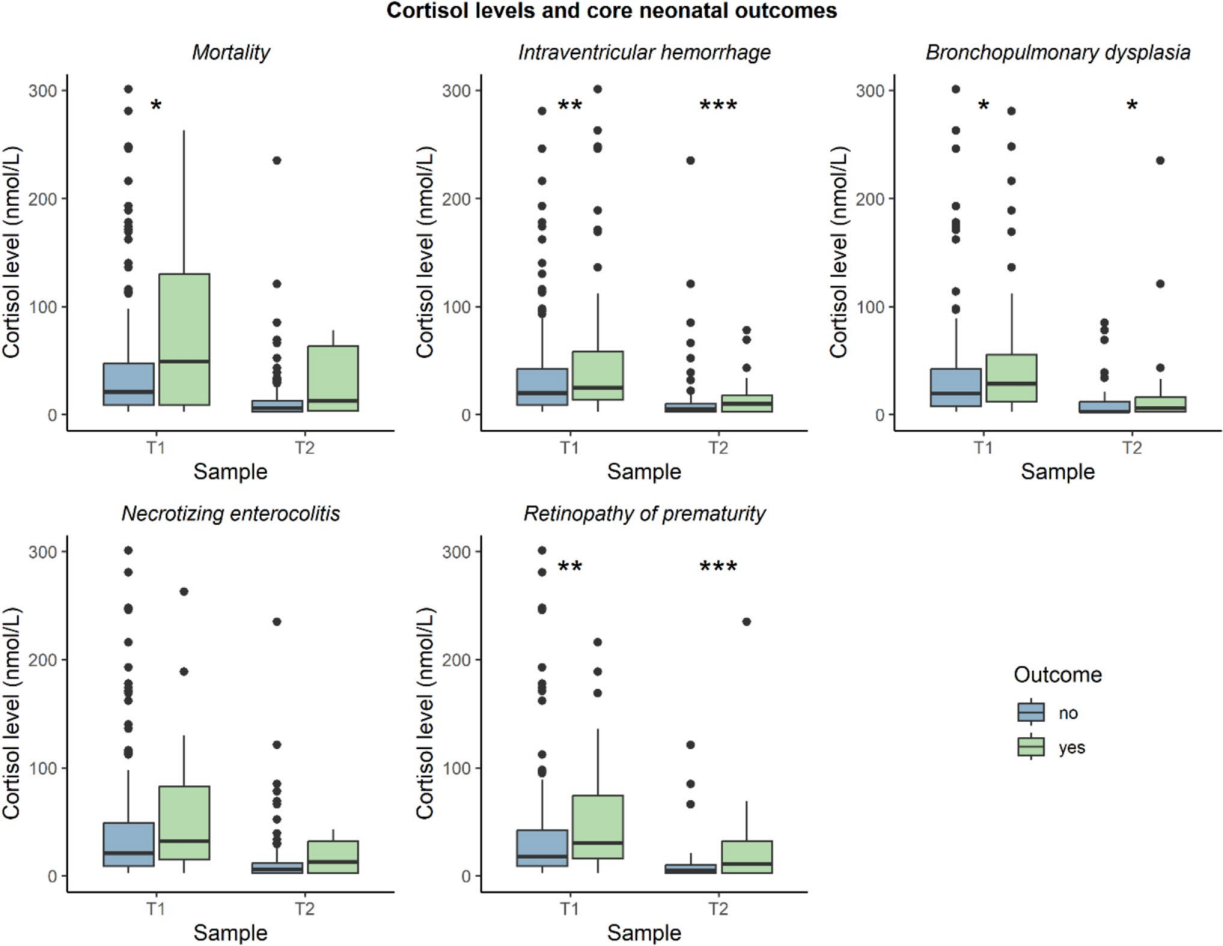
Urinary cortisol levels per time point and the occurrence of core neonatal outcomes. Cortisol levels were compared between those with and without the outcome using Mann–Whitney U tests. *P*-values below 0.05 are indicated with *, p-vales below 0.01 with **, and *p*-values below 0.001 with ***

Neonates who died had significantly higher cortisol levels at T1 than survivors (Fig. 3). Cortisol levels were higher at both T1 and T2 in neonates who developed IVH, BPD or ROP compared to those who did not. Cortisol levels did not differ significantly between those that did and did not develop NEC.

####  Multivariable logistic regression analyses

Multivariable logistic regression found no significant associations between cortisol levels at T1 or T2 and any of the morbidity outcome measures (Table 3). Stratified analyses in neonates with and without postnatal corticosteroid treatment found no associations between cortisol levels and BPD either.

**Table 3:** Association between cortisol levels at T1 and T2 and core neonatal outcomes

	Mortality			IVH			BPD			NEC			ROP		
	*β*	*95% CI*	*P-value*	*β*	*95% CI*	*P-value*	*β*	*95% CI*	*P-value*	*β*	*95% CI*	*P-value*	*β*	*95% CI*	*P-value*
Cortisol at T1															
Intercept	9.4			10			23			5.7			24		
Cortisol	−0.000072	−0.00048; 0.00025	0.69	−0.00012	−0.00048; 0.00014	0.42	0.000039	−0.00026; 0.00035	0.79	0.00040	0.000036; 0.00083	0.06	0.00048	0.000063; 0.0010	0.07
Female sex	0.36	−0.66; 1.4	0.48	−0.35	−0.87; 0.17	0.19	−0.17	−0.77; 0.43	0.59	−0.35	−1.3; 0.52	0.44	−0.71	−1.5; 0.037	0.07
Gestational age	−0.52	−0.88; −0.18	**0.004**	−0.40	−0.61; −0.20	** < 0.001**	−0.79	−1.07; −0.53	** < 0.001**	−0.33	−0.66; 0.0040	0.05	−0.96	−1.3; −0.67	** < 0.001**
Birth weight Z-score	0.0082	−0.039; 0.058	0.74	0.011	−0.015; 0.038	0.39	−0.070	−0.11; −0.037	** < 0.001**	−0.0095	−0.052; 0.034	0.66	−0.057	−0.095; −0.021	**0.002**
Single room NICU	1.1	−0.011; 2.3	**0.05**	0.29	−0.31; 0.89	0.33	0.29	−0.42; 0.98	0.42	−1.4	−2.9; −0.19	**0.04**	0.42	−0.40; 1.2	0.30
NEC	2.7	1.6; 3.9	** < 0.001**	0.26	−0.60; 1.1	0.54	−1.8	−3.0; −0.59	**0.004**				0.37	−0.83; 1.5	0.54
FIP	1.4	−0.38; 3.1	0.10	0.34	−0.97; 1.7	0.61	1.4	−0.33; 3.5	0.14	0.089	−1.6; 1.5	0.91	−0.049	−1.9; 1.8	0.96
Inotropic support	−0.0028	−1.1; 1.0	0.99	0.59	−0.10; 1.3	0.09	1.3	0.42; 2.2	**0.005**	1.7	0.86; 2.6	** < 0.001**	0.44	−0.44; 1.3	0.32
Intubated	0.88	−0.40; 2.1	0.16	−0.38	−1.2; 0.38	0.34	−0.47	−1.4; 0.48	0.34	−0.48	−1.9; 0.69	0.45	0.37	−0.66; 1.4	0.48
Antenatal steroid course: complete	0.24	−1.8; 3.4	0.85	−0.50	−1.5; 0.54	0.33	−1.7	−3.2; −0.31	**0.02**	0.53	−1.4; 3.6	0.65	0.60	−0.89; 2.4	0.46
Antenatal steroid course:.incomplete	1.0	−1.0; 4.2	0.41	−0.094	−1.1; 0.97	0.86	−2.1	−3.6; −0.63	**0.005**	0.65	−1.3; 3.7	0.58	0.26	−1.3; 2.1	0.76
Cortisol at T2															
Intercept	8.8			7.2			15			−3.0			25		
Cortisol	−0.00034	−0.0024; 0.0010	0.66	0.00015	−0.00065; 0.0010	0.71	0.00072	−0.00046; 0.0027	0.34	0.00022	−0.0019; 0.0018	0.81	−0.000067	−0.00095; 0.00087	0.88
Female sex	0.77	−0.80; 2.4	0.33	−0.30	−0.97; 0.35	0.37	−0.58	−1.3; 0.12	0.11	−1.2	−2.9; 0.067	0.09	−0.66	−1.5; 0.16	0.12
Gestational age	−0.51	−1.2; 0.069	0.096	−0.29	−0.55; −0.041	**0.02**	−0.54	−0.83; −0.26	** < 0.001**	0.018	−0.40; 0.45	0.93	−1.02	−1.4; −0.67	** < 0.001**
Birth weight Z-score	0.0056	−0.073; 0.084	0.89	0.036	0.0040; 0.071	**0.03**	−0.054	−0.092; −0.018	**0.004**	0.024	−0.029; 0.079	0.38	−0.066	−0.11; −0.025	**0.002**
Single room NICU	0.38	−1.9; 2.2	0.70	0.49	−0.27; 1.2	0.20	0.26	−0.54; 1.1	0.52	−18	−460; 48	0.99	−0.053	−1.0; 0.89	0.91
NEC	1.3	−0.62; 3.2	0.17	0.12	−0.96; 1.2	0.83	−0.41	−1.7; 0.85	0.53				1.75	0.42; 3.1	**0.01**
FIP	−0.52	−4.0; 2.1	0.72	−0.36	−1.9; 1.1	0.62	1.2	−0.54; 3.3	0.21	−0.14	−1.9; 1.4	0.87	0.29	−1.4; 2.1	0.74
Inotropic support	1.1	−0.54; 2.7	0.18	0.67	−0.089; 1.4	0.08	0.43	−0.42; 1.3	0.33	2.0	0.90; 3.2	** < 0.001**	0.64	−0.26; 1.5	0.16
Intubated	1.7	0.089; 3.3	**0.03**	−0.51	−1.5; 0.37	0.27	0.32	−0.70; 1.4	0.55	0.34	−1.2; 1.7	0.63	0.35	−0.77; 1.4	0.53
Antenatal steroid course: complete	−0.19	−2.7; 3.1	0.89	−0.24	−1.3; 0.91	0.68	−0.067	−1.5; 1.4	0.93	−0.21	−2.0; 1.9	0.83	1.34	−0.23; 3.2	0.12
Antenatal steroid course: incomplete	0.78	−1.8; 4.2	0.59	−0.10	−1.2; 1.1	0.86	−0.34	−1.8; 1.2	0.66	0.030	−1.8; 2.2	0.98	0.83	−0.81; 2.7	0.34

IVH, intraventricular hemorrhage; BPD, bronchopulmonary dysplasia; NEC, necrotizing enterocolitis; ROP, retinopathy of prematurity; FIP, focal intestinal perforation

## Discussion

This multicenter cohort study investigated the effects of stress exposure on the quantified stress responses, assessed by urine cortisol levels, in preterm neonates during their NICU admission. After the first postnatal week (T1), urinary cortisol levels were generally higher than after four weeks (T2), concurrent with a decrease in stress exposure in neonates born at a gestational age ≥ 26 weeks as measured by NeO-stress scores (Fig. 1) [26]. Neonates born at lower gestational ages typically experienced higher and more sustained stress exposure and had the highest cortisol levels. However, unlike more mature neonates, the most extreme preterm neonates lacked a positive association between stress exposure and cortisol levels at T1. By T2, cortisol levels tended to rise with stress exposure across all gestational ages. High cortisol levels were linked to neonatal morbidity and mortality, though not independently, with both cortisol levels and outcomes importantly influenced by gestational age.

Our findings that cortisol levels are highest in the youngest preterm neonates and decrease after the first postnatal week align with previous studies measuring cortisol in serum, plasma, and saliva [15, 29–31]. Similar to our results, one study found that the association between stress exposure and skin cortisol levels varied by gestational age, with a negative association in the most preterm and a positive association in more mature neonates [19]. In line with this, a study in moderately preterm infants found a positive association between Neonatal Infant Stressor Scale (NISS) scores and salivary cortisol levels [21]. Contrarily, a study among very preterm infants found no significant correlation between NISS scores and salivary cortisol levels [15]. However, they did not account for possible effect modification by gestational age.

The generally higher cortisol levels in the more preterm born neonates in our study likely reflect their greater stress exposure and critical illness. However, among the most extremely preterm neonates – who experience the highest stress – similar stress exposure corresponded to widely varying cortisol levels (Supplementary Fig. 4), and at T1, cortisol decreased with increasing stress exposure (Fig. 2). This suggests a subgroup of extremely preterm neonates with relative adrenal insufficiency (low cortisol despite high stress), resulting from adrenal immaturity and possibly persistence of fetal regulatory mechanisms [20, 25]. The inability of these neonates to mount adequate cortisol responses early in life could explain the negative stress-cortisol association at T1 in the most preterm neonates, especially since the sickest preterm neonates seem most vulnerable to relative adrenal insufficiency [32]. The high cortisol levels found in some extremely preterm neonates may reflect appropriate responses to the high stress they experienced, but may also be due to a lack of feedback control within the HPA axis [30]. Furthermore, differences in renal function – which matures postnatally in preterm infants – may play a role.

Although antenatal and postnatal corticosteroid treatments may also influence cortisol levels, these factors were adjusted for and thus cannot explain gestational age-dependent differences. The absence of a significant interaction between NeO-stress score and gestational age at T2 suggests that, by this time, HPA axis function in extremely preterm neonates may have matured. Moreover, many neonates had undetectably low cortisol levels at T2, making it challenging to detect associations with stress exposure.

Cortisol levels at T1 were higher in non-survivors than survivors, and at both time points were higher in neonates who developed IVH, BPD, or ROP compared to those who did not. These findings align with previous studies linking high cortisol levels to neonatal mortality and morbidity [20, 33–35]. However, after adjusting for clinical confounders, no significant associations remained, suggesting cortisol is more likely a marker of disease severity and degree of prematurity than a causal factor, although the observational nature of this study precludes drawing conclusions regarding causality. In the case of BPD, the association appears more complex, as low cortisol has also been associated with BPD [8], and the association may be influenced by postnatal corticosteroid treatment. However, subgroup analyses in neonates with and without postnatal corticosteroid treatment yielded similar findings, with no significant associations between cortisol and BPD. Possibly, cortisol precursor levels relative to cortisol are more informative for BPD risk [36].

A major strength of this study is its large, multicenter design with daily stress exposure estimates and cortisol measurement at two key time points. A limitation is that urine was collected at specific times rather than over 24-h, though this likely had little impact since preterm neonates lack a circadian cortisol rhythm in the first month [4]. Due to logistical challenges and early discharge, urine samples were not available for every neonate at both time points. More frequent sampling might have provided additional insight into basal cortisol levels, but the two standardized time points still allow meaningful group-level conclusions. Cortisol levels were not creatinine-corrected, which could improve measurement reliability, though particularly relevant for urine samples collected during the immediate postnatal period [37]. Stress assessment with the NeO-stress score did not include environmental stressors. Exact dates of neonatal outcomes were not recorded, limiting temporal analyses between cortisol and outcomes. As some conditions (e.g., IVH) occur early and others (e.g., BPD, ROP) develop later, fixed sampling times may not fully capture these associations. Finally, statistical power was limited for relatively rare outcomes such as NEC.

Both inappropriately low and excessive cortisol levels can be clinically relevant. Inappropriately low cortisol levels (relative adrenal insufficiency) have been linked to cardiovascular instability and BPD [25], and these neonates may benefit from cortisol replacement [38]. Conversely, high cortisol levels during critical windows may impair neurodevelopment [39, 40]. Additionally, early life stress may lastingly program HPA axis function, potentially affecting psychosocial development and cardiovascular health later in life [12]. Therefore, the extremely high cortisol levels found in some warrant caution. Further research is needed to determine the associations between very preterm neonates’ cortisol levels and long-term developmental and cardiovascular outcomes. Before cortisol can be used as a biomarker in the NICU, (noninvasive) measurement methods should be optimized [3] and proposed reference ranges validated [29].

Our findings provide insight into HPA axis development in preterm neonates admitted to the NICU, who experience high stress throughout the neonatal period. In more mature neonates, urinary cortisol levels may serve as an indicator of stress exposure; however, in the most preterm neonates, wide variability despite comparable stress exposure limits cortisol’s utility as a stress marker. A deeper understanding of preterm neonates’ stress responses may guide the development of targeted stress reduction strategies and identify neonates at risk as neonates with inappropriately low cortisol levels that may benefit from hydrocortisone treatment. Reducing stress during NICU admission is urgently needed, as stress in preterm neonates is associated with various detrimental effects, for instance on cognitive and emotional development [12]. Approaches to reduce stress in the NICU include adequate pain management, minimizing painful and stressful procedures, increasing parental presence, touch and voice, and environmental control (reducing noise and excessive lights).

## Conclusions

Urinary cortisol levels in very preterm neonates are affected by stress exposure. After the first postnatal week, this relationship between stress exposure and cortisol levels is moderated by gestational age, with more mature neonates showing greater cortisol responses to stress exposure, probably caused by relative adrenal insufficiency in the most preterm born infants. Neonates who died or developed IVH, BPD or ROP had higher cortisol levels than those who did not, but cortisol levels were not independently associated with these outcomes. These results provide insight into HPA axis function in very preterm neonates, which may guide the development of stress reducing interventions.

## Supplementary Information

Below is the link to the electronic supplementary material.

Supplementary file1 (PDF 290 KB)

## Data Availability

The data that support the findings of this study are available from the corresponding author upon reasonable request.

## Abbreviations

BPD: Bronchopulmonary dysplasia
CI: Confidence interval
HPA: Hypothalamic-Pituitary-Adrenal
IVH: Intraventricular hemorrhage
NEC: Necrotizing enterocolitis
NICU: Neonatal Intensive Care Unit
ROP: Retinopathy of prematurity
