# Supplementary material for: Stress exposure, stress responses, and short-term outcomes in very preterm neonates: a national cohort study
Source: Eur J Pediatr. 2026 Feb 11;185(2):129. doi: 10.1007/s00431-026-06765-1 (PMC12894146; doi:10.1007/s00431-026-06765-1)

Supplementary figure 1. Flowchart of the included participants.

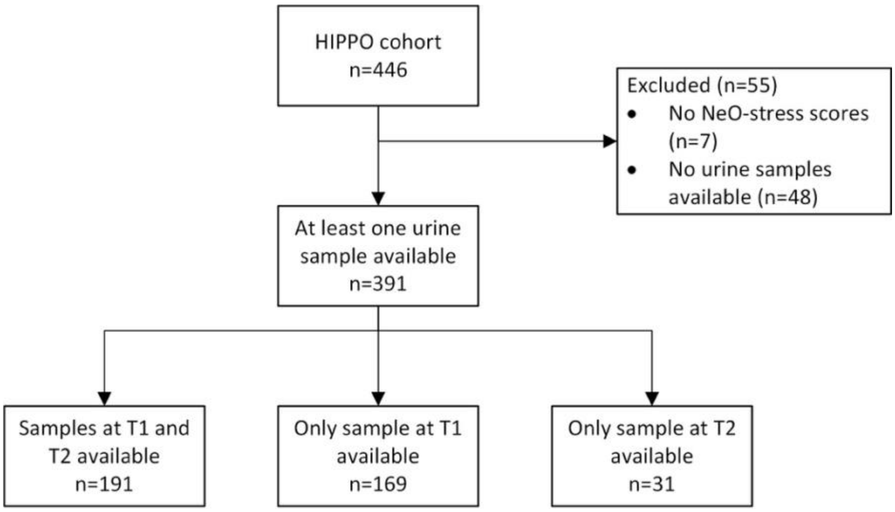

Supplementary figure 2. Boxplot of corticosteroid levels at sampling moment T1 and T2. Twenty participants had very high cortisol measurements exceeding 1000 nmol/L, which have been omitted from this figure. Cortisol-G measurement often failed. At T1, 30 (8%) neonates had undetectably low (< 5 nmol/L) cortisol levels, and at T2, 93 (42%) neonates had undetectably low cortisol levels.

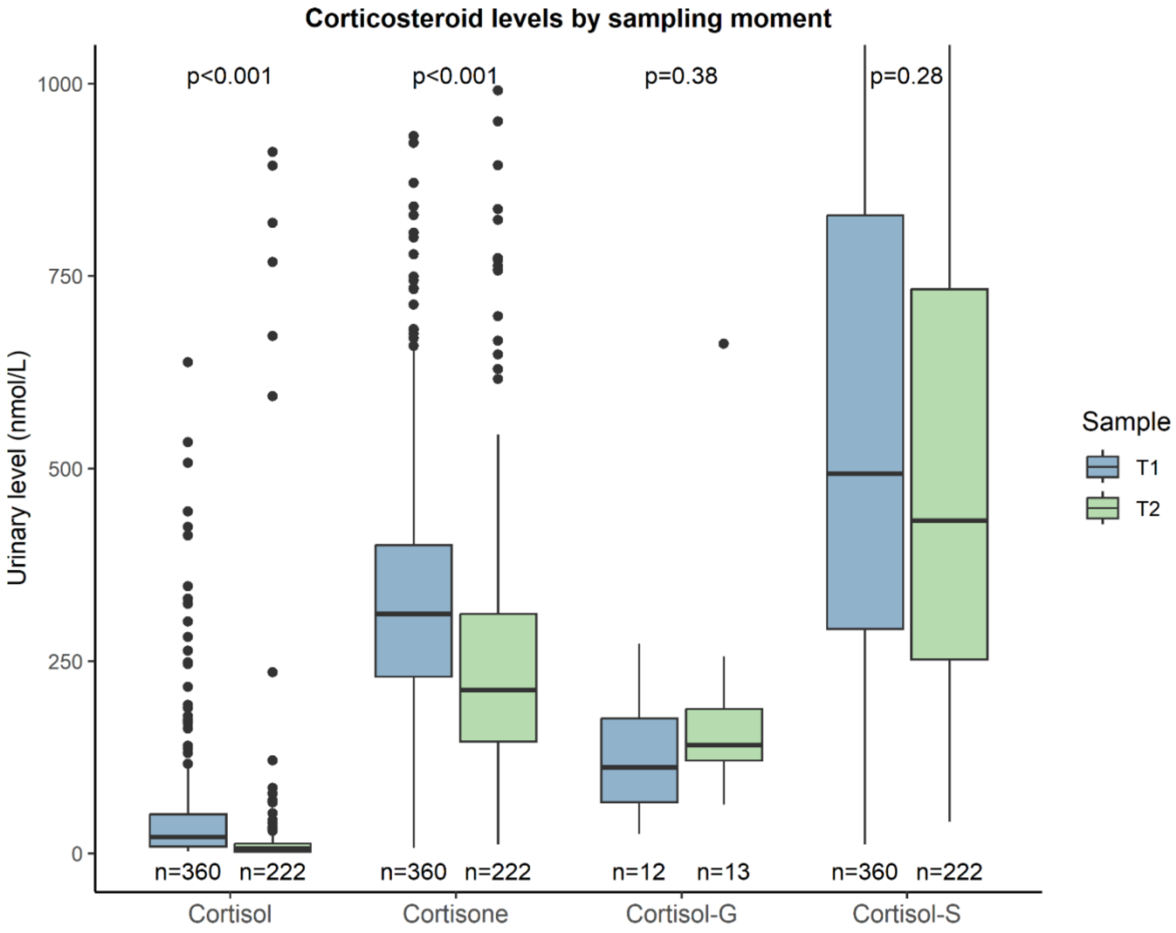

Supplementary figure 3. Spaghetti plot illustrating participants' cortisol levels and corresponding cortisone levels at T1 and T2.

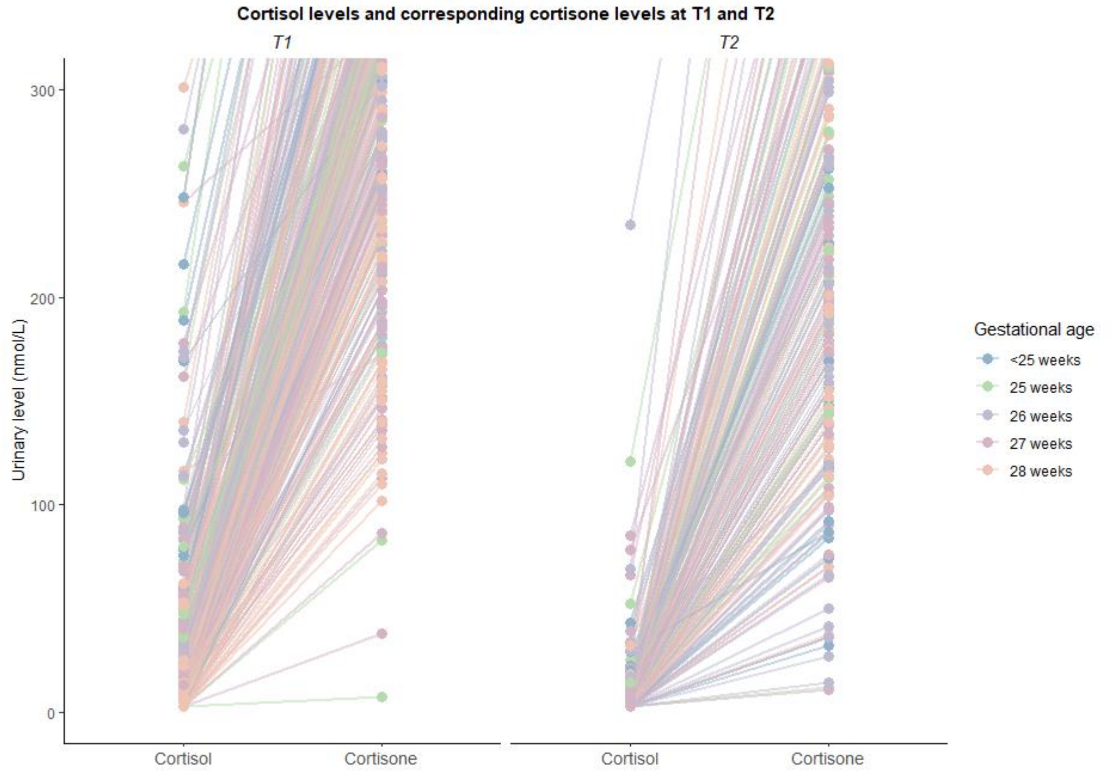

Supplementary figure 4. Scatterplot illustrating the relationship between average NeO-stress score (in all days preceding urine collection) and urinary cortisol levels, stratified by gestational age group. Each dot indicates a participant. The overlying ellipses represent 95% confidence regions for each group, calculated using a t-distribution, providing a visual summary of the relationship and variability within each group.

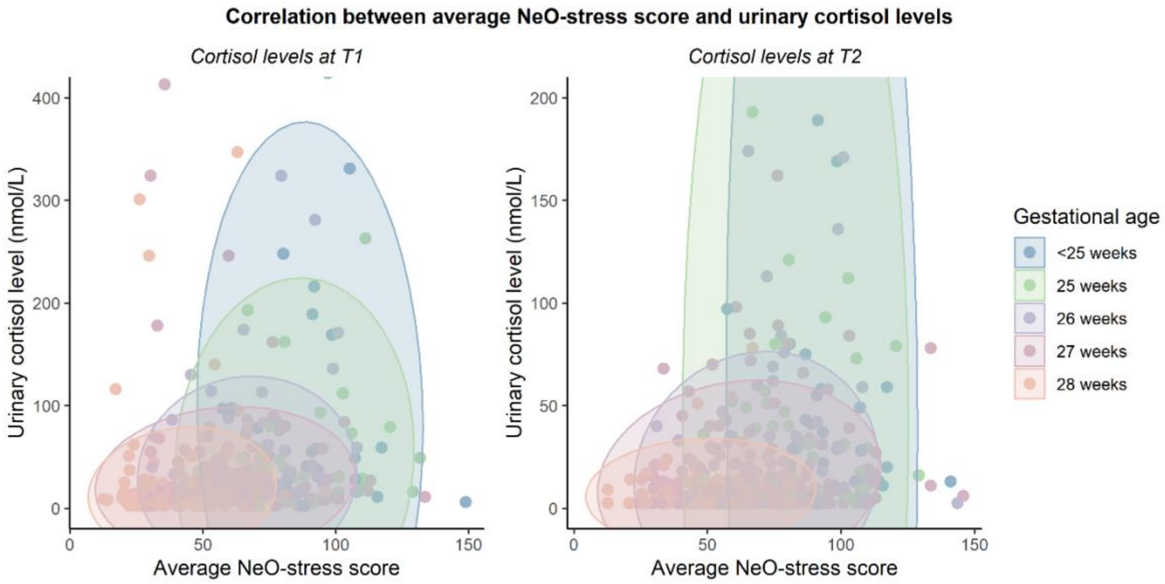

Supplement: Supplementary file 1 — Supplementary file1 (PDF 290 KB) [file 431_2026_6765_MOESM1_ESM.pdf]
